# Inosine Incorporation in DNA Nanostructures and 3D DNA Crystals

**DOI:** 10.1021/acs.nanolett.6c02772

**Published:** 2026-09-02

**Authors:** Arun Richard Chandrasekaran

**Affiliations:** † Department of Nanoscale Science and Engineering, University at Albany, State University of New York, Albany, New York 12222, United States; ‡ The RNA Institute, University at Albany, State University of New York, Albany, New York 12222, United States

**Keywords:** DNA nanotechnology, inosine, noncanonical bases, DNA self-assembly

## Abstract

DNA nanotechnology is based on programmable base pairing, resulting in the precise construction of nanoscale structures. Sequence variability in DNA self-assembly is achieved by the use of xenonucleic acids, chemically modified bases, and base analogues. The naturally occurring base inosine, while well-studied in RNA editing, has not been used in the context of DNA nanotechnology. Here, I demonstrate the use of inosine in DNA nanostructures, specifically by incorporating inosine within the duplex regions or junctions of a double-crossover DNA motif. In strand displacement and competition assays, canonical complements do not displace inosine-containing strands postassembly but dominate in product formation when competing with inosine-containing strands during assembly. Finally, sticky ends with inosine base pairs enable the formation of rationally designed 3D DNA crystals based on the tensegrity triangle motif. Overall, this work shows that inosine is a useful addition to the library of sequence variations in DNA nanotechnology.

## Graphical abstract

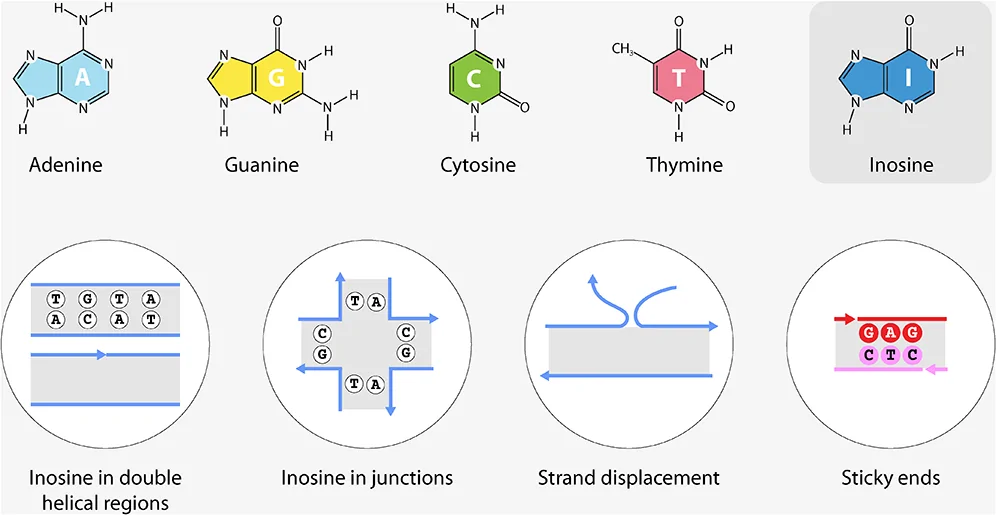

DNA nanotechnology is based on the principles of programmable base pairing, with precise construction of structures ranging from the nanometer to the micrometer scale.^1–4^ Self-assembled DNA nanostructures are useful in biosensing, drug delivery, photonics, and data storage, and as scaffolds for biomolecular encapsulation.^5–10^ A key factor in DNA-based construction is the exploration of the sequence landscape for better nanoscale assembly. Specific base-pairing rules have been developed for DNA motifs,^11–14^ sequence symmetry minimization is followed to avoid undesired structures,^15,16^ and multifold repeating sequences are used to create DNA nanostructures with fewer component strands.^17,18^ Sequence choices that prevent base pairing are also useful, such as in the use of poly-T tails and blunt-ended interactions, to avoid unnecessary stacking of structures leading to aggregates.^19–22^ In most of these cases, the component sequences are based on the canonical Watson–Crick–Franklin base pairs. Some recent studies, including our own, have indicated that nanostructure stability and functionality can be sensitive to even single-nucleotide mismatches and the type of base stack at nick sites.^23–25^ Thus, while advances in the construction of complex objects and devices using DNA are significant for applications, studies on the underlying base pairs and their effect on DNA self-assembly contribute important information to create DNA nanostructures.

Beyond the traditional Watson–Crick–Franklin base pairing, the use of noncanonical base pairing, modified bases, and DNA analogues has also been demonstrated in DNA nanotechnology.^26–28^ Noncanonical nucleic acids with natural or synthetic structural modifications exhibit enhanced stability and unique functional potential.^29,30^ For example, the wobble base pair G:U is used to mitigate secondary structures in some nanostructures,^31^ and the base pair A:C is used in creating RNA nanostructures with only three bases (A, C, G).^32^ In addition, metal-mediated base pairing has also been used to strengthen mismatches within DNA nanostructures, such as C–Ag^+^–C^33^ and T–Hg^2+^–T.^34^ Unnatural base pairs such as 5-Me-isoC/isoG and A/2-thioT have also been incorporated into 6-arm DNA junctions and 6-helix nanotubes, resulting in enhanced thermal stability and resistance to T7 exonuclease digestion.^35^ Xenonucleic acids such as threose nucleic acid,^36^ acyclic threoninol nucleic acid,^37^ glycerol nucleic acid,^38^ and peptide nucleic acid^39^ have also been used in DNA-based construction. Recently, newer DNA base analogues have also been created and their use has been demonstrated in DNA nanotechnology.^40,41^ For example, Z:P base pairs were incorporated into the double-helical domains of tensegrity triangle motifs that yielded 3D crystals,^40^ and these synthetic bases were also used in triplex-forming oligonucleotides (TFOs) to target specific duplexes.^41^ Further, other purine analogues (e.g., B:P and D:X) and pyrimidine analogues (e.g., S:Z and T:K) have been successfully used in several extended tile-based 2D arrays.^42^ The incorporation of such unnatural base pairs in DNA nanostructures provides an additional sequence library as well as varied stability and functionality with enzymes such as polymerases and nucleases.^43^

However, a common base, inosine, has not been incorporated into DNA nanostructures, and its effect on the self-assembly and properties of such structures has not been explored systematically. Inosine arises by the deamination of adenosine where an amine group is replaced by a carbonyl (Figure 1a). Inosine behaves like guanosine during base pairing, preferentially forming hydrogen bonds with cytidine. I:C base pairs have reduced stacking energy and form two hydrogen-bond interactions, rather than three in G:C base pairs. Further, poly I:C has been shown to form B-DNA similar to the right-handed B-DNA form of the canonical poly A:T or poly G:C stretches.^44^ Moreover, inosine does not destabilize short DNA helices when it pairs with C, U, or A,^45,46^ with the stabilities of inosine base pairs being G:C > I:C > I:A > I:G ≈ I:T (Figure 1b).^47,48^ In RNA, A-to-I editing introduces an A-to-G substitution in the RNA sequence without altering the genomic sequence. The effect of G-to-I substitution in A-RNA and B-DNA has been shown to depend on the different topologies of these helices, with the different electrostatic interactions between the base pairs and the negatively charged backbone playing a role.^49^ The broad base-pairing potential of inosine is also naturally observed in cytosolic tRNAs where an inosine is located at the 5′ position (wobble position) of the anticodon,^50^ pairing with C, U, or A as originally proposed by Crick.^51^ This base-pairing potential, structural similarity to B-DNA when paired, and the varied functionality of inosine make it highly suitable for use in nucleic acid nanotechnology.

**Figure 1:**
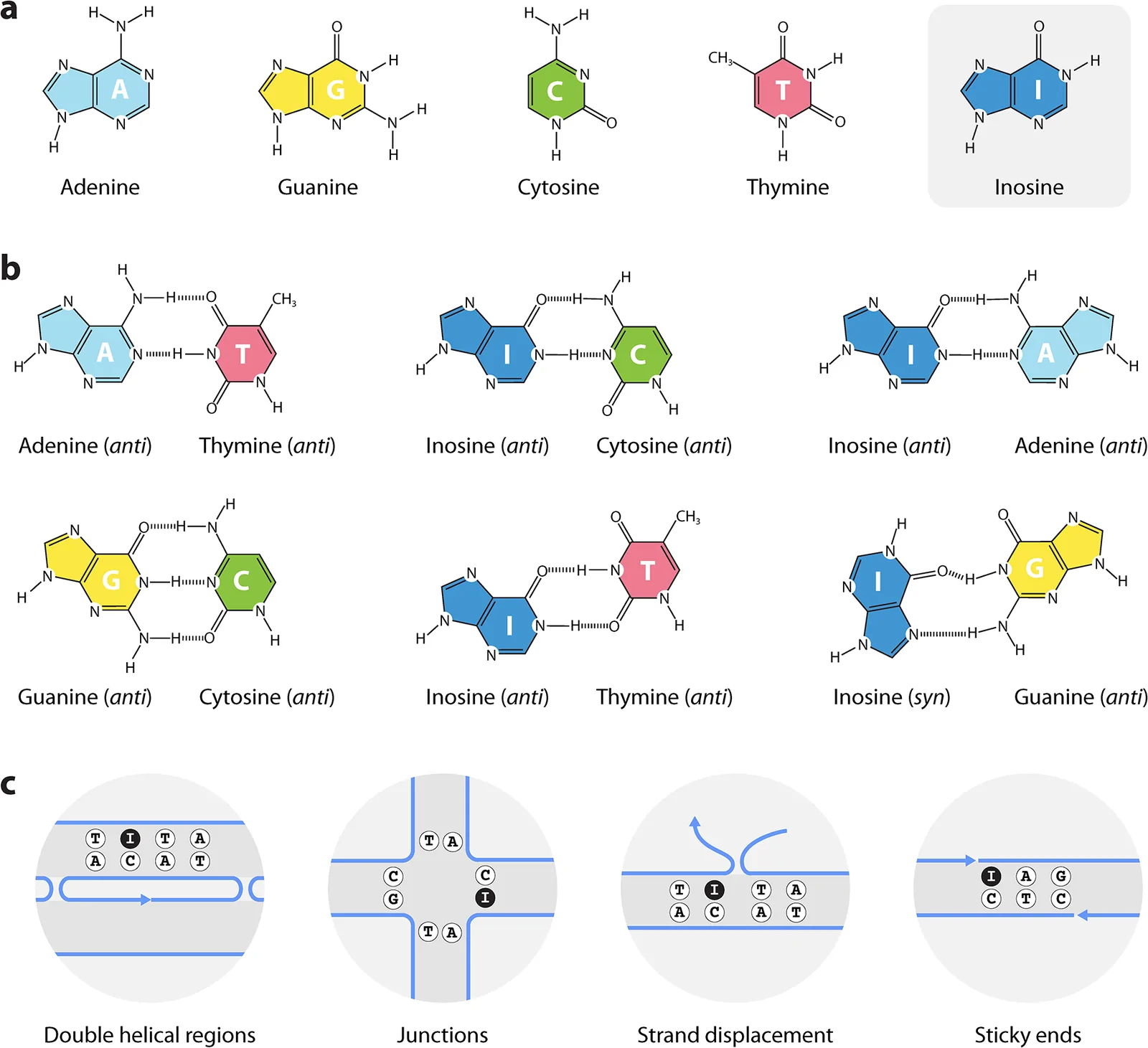
Inosine base pairs. (a) Chemical structures of DNA bases adenine (A), guanine (G), cytosine (C), thymine (T), and inosine (I). (b) Canonical A:T and G:C base pairing (left) and inosine base pairs (right). (c) This study explores inosine incorporation within double-helical edges and junctions of DNA nanostructures, the effect of inosines in strand displacement, and inosine incorporation in sticky ends for hierarchical self-assembly.

Here, I present the effect of inosine incorporation in DNA nanostructures using a model DNA motif: the double-crossover (DX) motif. Specifically, inosines were incorporated in the duplex region between the crossovers in the structure or at the junction points where the two crossovers occur. In addition, I study the competition between inosine-containing strands and strands containing perfect base pair matches in duplex assembly, as well as toeholdless strand displacement involving inosine base pairs. Using the tensegrity triangle motif, I also demonstrate efficient sticky end cohesion with inosine base pairs in the hierarchical self-assembly of rationally designed 3D DNA crystals.

To study the effect of inosine incorporation in DNA nanostructures, I first used the DX DNA motif,^52^ testing incorporation in two structural regions: one of the double-helical regions between the crossovers and within the two junctions. The DX motif has 21 bp between the crossovers, allowing testing of modifications in a 2-turn double-helical region constrained by a junction at either end (Figure 2a, Figure S1 and Table S1). I modified the contiguous strand to contain 1, 2, 3, or 4 inosines, with the inosines separated by two bases between them. The DX structures containing a different number of inosines can thus be assembled by changing only one of the five component strands. Nondenaturing polyacrylamide gel electrophoresis showed proper assembly of the structures in 1× TAE with 12.5 mM Mg^2+^, with an ∼10% decrease in assembly yield for the structure with 4 inosines compared to the control structure without any inosines (Figure 2b and Figure S2). Thermal melting analysis reflected these results, with a slight monotonic reduction in melting temperature as the number of inosines was increased, with an ∼3.5 °C difference between the control structure and the structure containing four inosines (Figure 2c and Table S2). The melting temperatures of the structures with 2 or 3 inosines were similar, possibly due to the specific base pairs involved in the inosine positions. Previous studies showed that the effect of inosine incorporation on reverse transcription has also been minimal, possibly due to the tolerance of inosine base pairs in RNA as in DNA observed here.^53^

**Figure 2:**
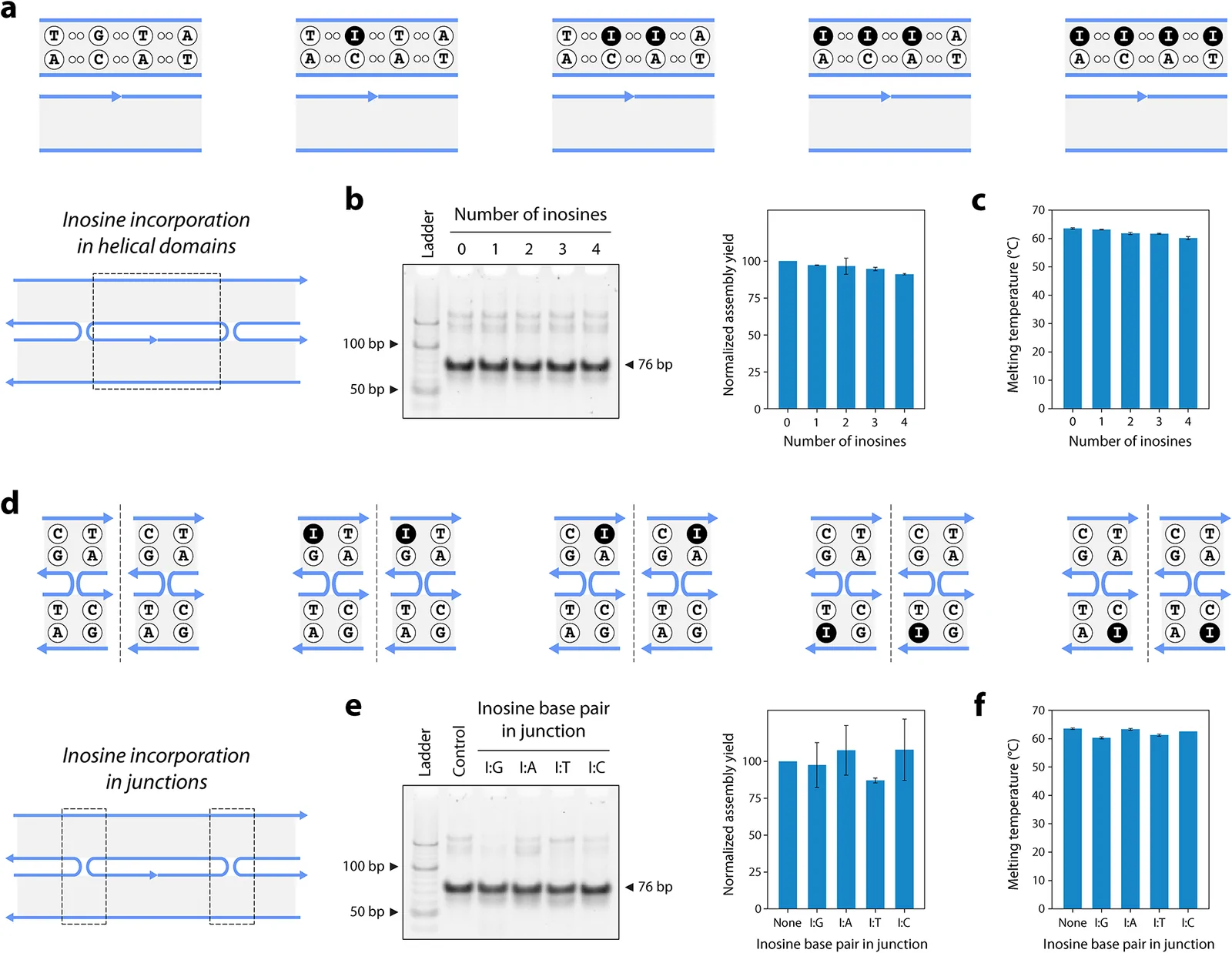
DX DNA motif containing inosines. (a) Schematic showing the double-helical edge of a DX DNA motif where inosines were incorporated. (b) Nondenaturing gel and assembly yields and (c) melting temperatures for the DX DNA motif with different numbers of inosines. (d) Schematic, (e) nondenaturing gel and assembly yield, and (f) melting temperatures for the DX DNA motif with different inosine base pairs at the two junctions.

Next, I studied the effect of inosines on the junctions of the DX motif. The sequence of the DX at the junction allows each type of base in the core four positions to be replaced by an inosine, i.e., I:G instead of C:G, I:T instead of A:T, I:C instead of G:C, and I:A instead of T:A (Figure 2d and Figure S3). This design allows the study of inosine incorporation within a DNA junction while also comparing the different effects on the four types of base pairs. Compared to the control structure without inosines, the assembly of the inosine-containing structures was similar, indicating a minimal effect when only one of the base pairs of the junction was modified (Figure 2e and Figure S2). The melting temperature for the structure containing the I:G base pair in the junction was slightly lower compared to the control structure and the structures with other inosine base pairs in the junctions (Figure 2f and Table S2).

I then studied the competition between strands containing the canonical base pairs and inosine base pairs pre- and postassembly. In one of our recent works,^24^ we demonstrated mismatch-induced strand displacement where the instability in a duplex due to mismatches allowed strand displacement to occur without the presence of a toehold. I tested a similar effect here to determine whether the inosine base pairs were less stable compared to the canonical base pairs, thereby allowing a full complement to displace it. I designed duplexes formed by strand X* and a complement containing 1, 2, 3, or 4 inosines (X-I_*n*_). To the assembled duplex, I added an invading strand with a perfect match to observe whether the inosine-containing strands are displaced. I included poly-T extensions at either end of the invading strand (X_T_) to allow convenient analysis by differential gel migration of the initial and final duplexes (Figure 3a and Figure S4). While there was a trend related to the number of inosines in the duplex, there was minimal strand displacement overall compared to the control structure (Figure 3b and Figure S5). This result indicates that postassembly, canonical base pairing did not efficiently replace existing inosine-containing base pairs in these duplexes even after 24 h, despite the reduction in thermal stability of the duplexes with an increasing number of inosines, with an ∼10 °C difference between the control and the duplex containing four inosines (Figure S6 and Table S3).

**Figure 3:**
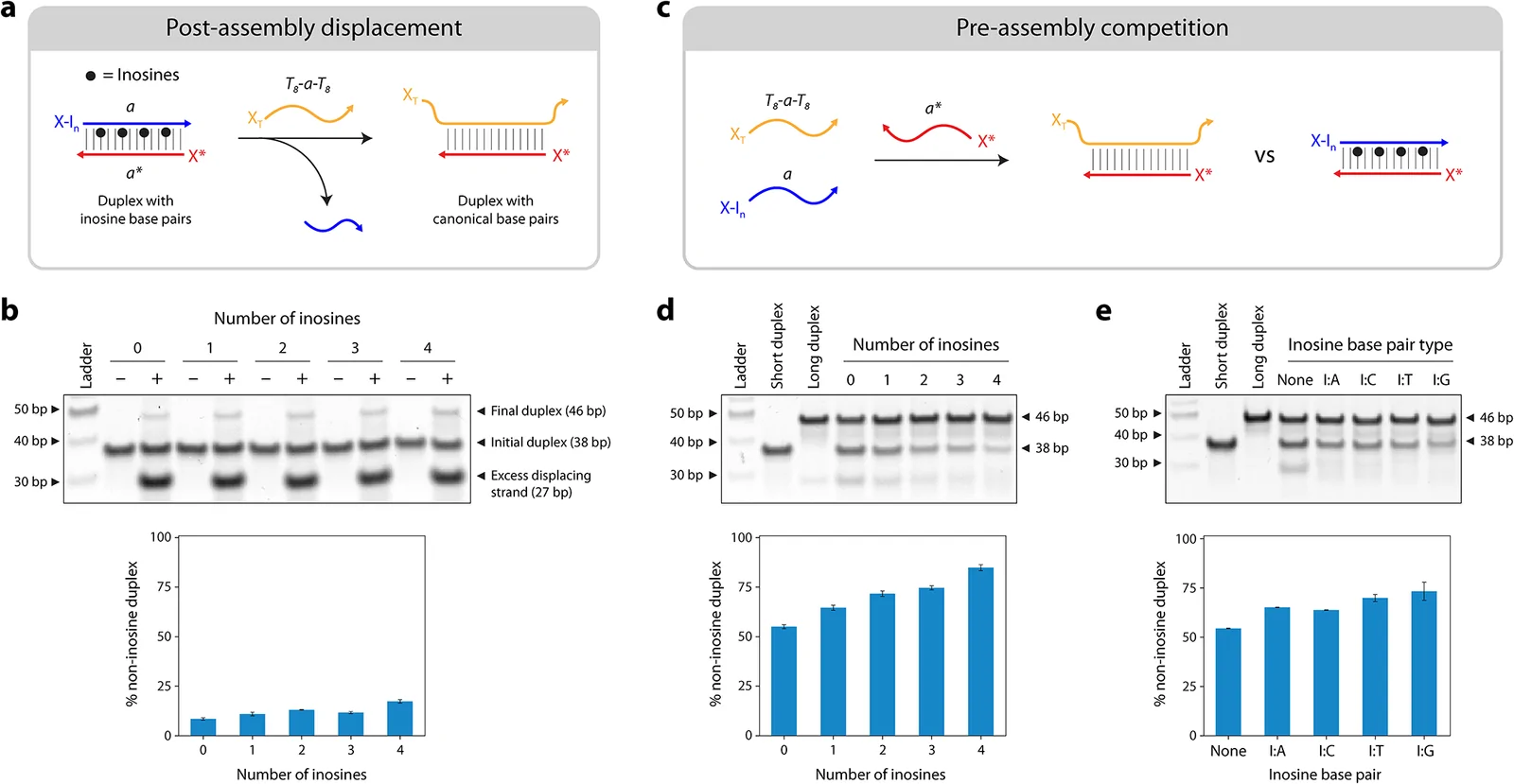
Strand displacement and competition. (a) Displacement of an inosine-containing strand from a preassembled duplex. The invading strand X_T_ contains a full complement to strand X* and additional Ts at the two termini to differentiate the final complex from the initial duplex on a gel. (b) Gel results and quantified displacement efficiencies. (c) When inosine-containing complement and a full conventional complement compete for the same binding partner (X*), the strand containing canonical bases is preferred. The strand containing a higher number of inosines is less preferred. (d) Gel results and quantified competition efficiency in strands containing different numbers of inosines. (e) Gel results and quantified competition efficiency in strands containing different types of inosine base pairs.

Based on this result, I then tested whether inosine-containing strands and canonical complements would compete in DNA duplex formation (Figure 3c). I mixed component strands with inosines (X-I_*n*_) and without inosines (X_T_), both of which are complementary to strand X*, and annealed the solution. Again, the canonical complement had poly-Ts on the termini to allow gel-based identification of the products. The control without inosines also showed 50% of each product due to the equilibrium between both complements, a feature we have observed in our earlier work.^24^ Results showed that in each case the complement with the canonical bases formed the major product, with up to ∼85% yield compared to only ∼15% yield for the duplex containing four inosines (Figure 3d and Figure S7). Notably the duplex product with the inosine-containing strand decreased as the number of inosines increased. In contrast to postassembly displacement where canonical base pairs did not displace inosine-containing strands from a premade duplex, the strand with conventional base pairs is preferred when directly competing with a complementary partner in solution.

In the above experiments, base-pairing preference between canonical base pairs and inosine base pairs was studied with an increasing number of inosines. To test the preference of specific base pairs over inosine, I then performed a similar preassembly competition experiment where the competing strand contained only an A, T, C, or G at specific positions in the strand where inosines were incorporated, allowing the comparison of A:T vs I:T, T:A vs I:A, C:G vs I:G, and G:C vs I:C (Figure S8). Similar to the result above, a competition experiment revealed the preference toward conventional base pairs, with a noticeable trend in the type of base pair preferred over an inosine-containing base pair (Figure 3e and Figure S7). Compared to the control without inosines, which showed 50% of each product, I:A and I:C base pairs showed ∼63–65% displacement, while the duplexes containing I:T and I:G showed ∼70–73% displacement, indicating slightly different efficiencies in competition within different types of inosine base pairs.

Next, I tested the incorporation of inosine in sticky ends that allow the hierarchical assembly of DNA nanostructures. As a model system, I chose three-dimensional (3D) DNA crystals self-assembled from the tensegrity triangle DNA motif, a system I’ve extensively worked on and that is well-studied by other groups as well.^54–68^ These studies have demonstrated the dictated self-assembly of DNA motifs into 3D DNA crystals, with chemical modifications that result in higher stability,^59^ larger crystal growth,^67^ and efficient TFO binding.^57^ The tensegrity triangle structure used here contains three double-helical edges (each 31 bp in this case) that are connected at the vertices by a four-arm junction (Figure 4a).^69^ The structure is inherently three-dimensional, with an over-and-under arrangement of the helical edges. The edges are further tailed by sticky ends that allow the triangles to connect to each other, resulting in the formation of a 3D DNA lattice. Typically, the triangles are tailed by 2 nucleotide sticky ends that result in crystal formation; however, other sticky end lengths and sequences have also been studied.^58^ I modified one of the sticky end bases to inosine to study whether inosine-containing sticky ends allow hierarchical assembly of DNA motifs, especially in this 3D arrangement where micrometer-sized crystals contain trillions of the starting triangle motif (Figure S9). There is only one occurrence of an inosine-containing sticky end in DNA nanostructures, where an II:CC sticky end was used in a similar crystal assembled from 2-turn tensegrity triangles (a study I was part of).^58^ Here, I use tensegrity triangle DNA motifs with 1-nt and 3-nt sticky ends containing inosines.

**Figure 4:**
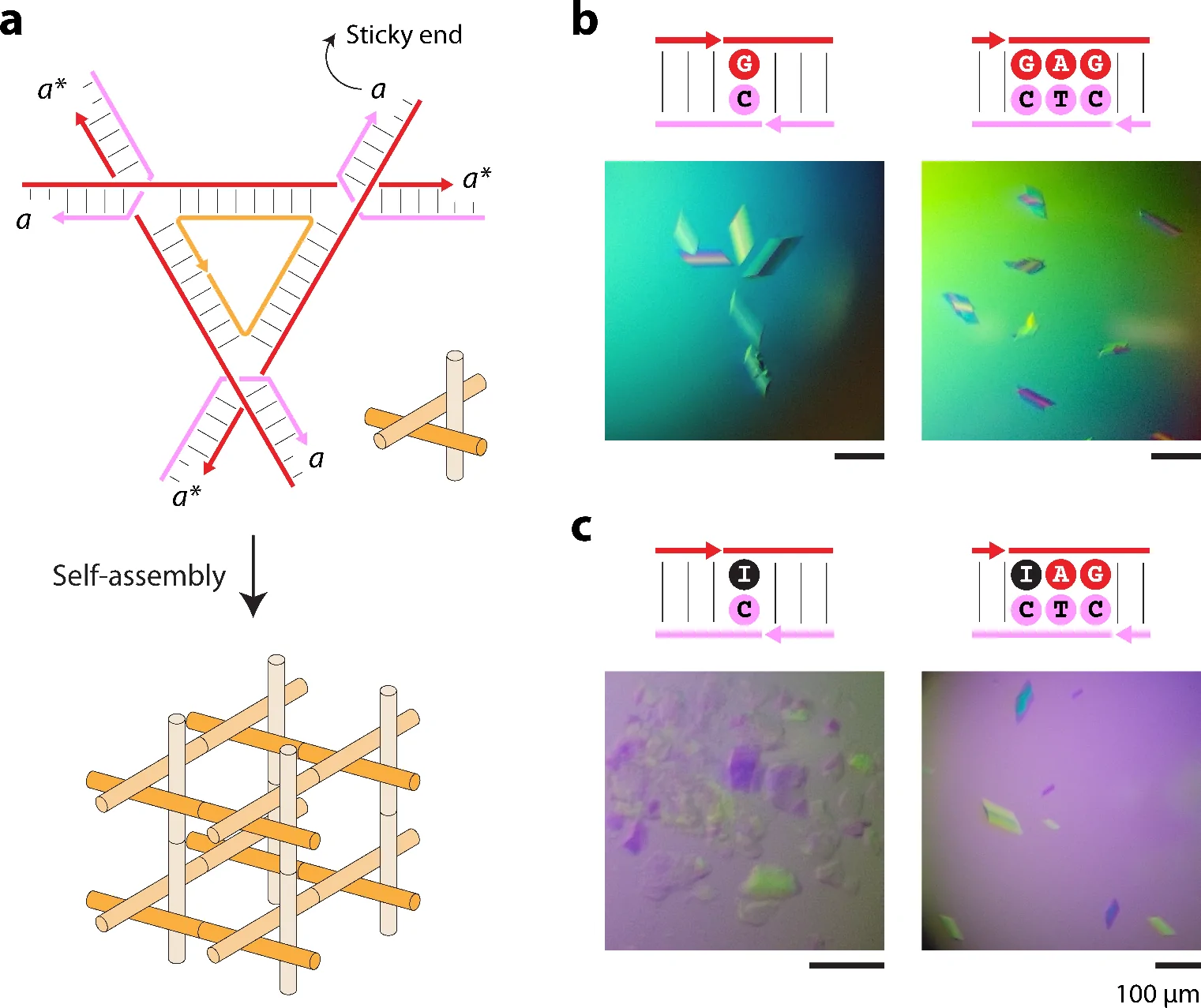
Hierarchical assembly of 3D DNA crystals. (a) Schematic of the tensegrity triangle motif and self-assembly via sticky end cohesion into 3D crystals. Each edge of the motif contains 31 bp regardless of the sticky end length. Crystals obtained from tensegrity triangle motifs using sticky ends without (b) and with inosine (c).

Nondenaturing gels showed proper assembly of each version of the triangle, with a single band corresponding to the structure (Figure S10). For the structure with a 3-nt GAG:CTC sticky end, I also observed additional bands in the well, a result that corresponds to stronger sticky end cohesion resulting in hierarchical structures, as these self-assembled crystals have been observed to form in solution in our prior work as well as reports by others.^67^ I set up crystals using the triangles with different sticky end lengths, both with and without inosines. I observed crystal growth in all of the triangular assemblies, indicating that the sticky ends allowed self-assembly of the triangles into crystals (Figure 4b). Inosine-containing sticky ends also allowed crystal formation, with the crystal habit observed resembling those of the controls and the typical shape observed for these designer DNA crystals in our previous studies (Figure 4c). The observation of crystals under a polarizer confirmed that the assemblies were crystalline in nature and not aggregates. In addition to the one prior report of an II:CC sticky end, I show in this work that even with a single nucleotide pair, where the interaction is I:C, the triangles are still able to self-assemble into 3D crystals.

Overall, this work shows that inosine is a useful addition to the library of sequence variations in DNA nanotechnology. Incorporation of inosines in the duplex region or junctions minimally affects DNA nanostructure assembly, which is a result that can be adapted for other structures. Further, understanding the role of inosine in strand displacement and competition reactions adds to the knowledge base on the influence of nucleotide types in DNA strand displacement.^27^ Another potential aspect of expanding the type of bases used in DNA nanostructures is the different biostabilities conferred by unconventional nucleotides, a feature that has yet to be studied for inosine.^70^ This study also had its limitations. First, the postassembly displacement and preassembly competition experiments comparing inosine base pairs with canonical base pairs are performed within the context of a specific set of duplexes. The site of inosine incorporation (middle vs terminus), the density (inosine placed together vs spread out), and the overall length of the duplex can all influence the effect of inosines on the stability of the structures. Second, the incorporation of inosine in junctions is within a DX motif that is stabilized by double-helical domains stitched together in the middle. Stand-alone junctions with shorter double-helical arms might be more sensitive to such sequence modifications. Third, while crystal assembly is successful, further X-ray diffraction studies are needed to ascertain changes to the unit cell parameters or any effect of inosines in the sticky end on the underlying structure in these crystalline assemblies. Inosine-induced base-pairing diversity in RNA is well-studied, but more studies are needed to understand the structural effects of inosine base pairs in DNA helices. Increasing use of inosines in DNA-related applications, such as the work reported here, may initiate more detailed studies on the topic.

## Supplementary Material



## Notes

Experimental procedures, additional results, and DNA sequences used (PDF)
