# Supplementary material for: Inosine Incorporation in DNA Nanostructures and 3D DNA Crystals
Source: Nano Lett. 2026 Sep 2;26(36):12070–7. doi: 10.1021/acs.nanolett.6c02772 (PMC13587659; doi:10.1021/acs.nanolett.6c02772)
Supplement: Supplementary file 1 [file nl6c02772_si_001.pdf]

Supporting information

Inosine incorporation in DNA nanostructures and 3D DNA crystals

Arun Richard Chandrasekaran[1,2,*]

[1]Department of Nanoscale Science and Engineering, University at Albany, State University of New York, Albany, NY, USA.
[2]The RNA Institute, University at Albany, State University of New York, Albany, NY, USA.

*Correspondence: arun@albany.edu

Methods

Preparation of DNA complexes. DNA strands were purchased from Integrated DNA Technologies (IDT). DNA complexes were annealed in TAE buffer containing 40 mM tris base (pH 8.0), 20 mM acetic acid, 2 mM EDTA and 12.5 mM magnesium acetate (concentrations in 1× buffer). DX samples were annealed in a thermal cycler with the following steps: 90 °C for 3 minutes, 65 °C for 20 minutes, 45 °C for 20 minutes, 37 °C for 30 minutes, 20 °C for 30 minutes, and the solution was then cooled to 4 °C. Duplexes were annealed from 90 °C to 20 °C over ~30 min. Tensegrity triangle nanostructures were annealed from 90 °C to 20 °C over 2 days.

Gel electrophoresis. Non-denaturing polyacrylamide gels were prepared using 19:1 acrylamide to bisacrylamide stabilized solution (National Diagnostics) in 1× TAE-Mg^{2+} buffer. DNA samples were mixed with loading dye containing bromophenol blue and glycerol before loading into gels. Gels were run at 4 °C in 1× TAE-Mg^{2+} running buffer and stained with 0.5× GelRed (Biotium) and destained in water. Gels were imaged on a Bio-Rad Gel Doc XR+ imager using the default settings for GelRed with UV illumination and analyzed using ImageLab software (Bio-Rad). The assembly yield was quantified as the fraction of the intensity of the band corresponding to the structure compared to the total intensity of all the bands in the lane. Data corresponding to gel analysis represent mean and standard deviation from replicate experiments.

Thermal melting studies. UV thermal melting experiments were performed in a Cary 3500 UV-Visible Spectrophotometer equipped with a temperature controller. Melting curves were acquired at 260 nm by heating and cooling from 20 °C to 90 °C at a rate of 0.5 °C/min for DX motifs and at a rate of 1 °C/min for duplexes. The data were fitted to the Boltzmann function using OriginPro. Melting temperatures were determined from the first derivative of the fitted melting curves. Data presented in graphs represent mean and standard deviation from replicate experiments.

Crystallization. 5-6 μl of annealed tensegrity triangle structures were equilibrated against a 600 μl reservoir of 1.75 M ammonium sulfate in hanging drops set up at 20 °C. Crystals were also set-up using the DNA solution in hanging drops and the entire set up placed in a thermal gradient from 60 °C to 20 °C. Crystals were imaged using a Zeiss SteREO Discovery V12 microscope fitted with a polarizer.

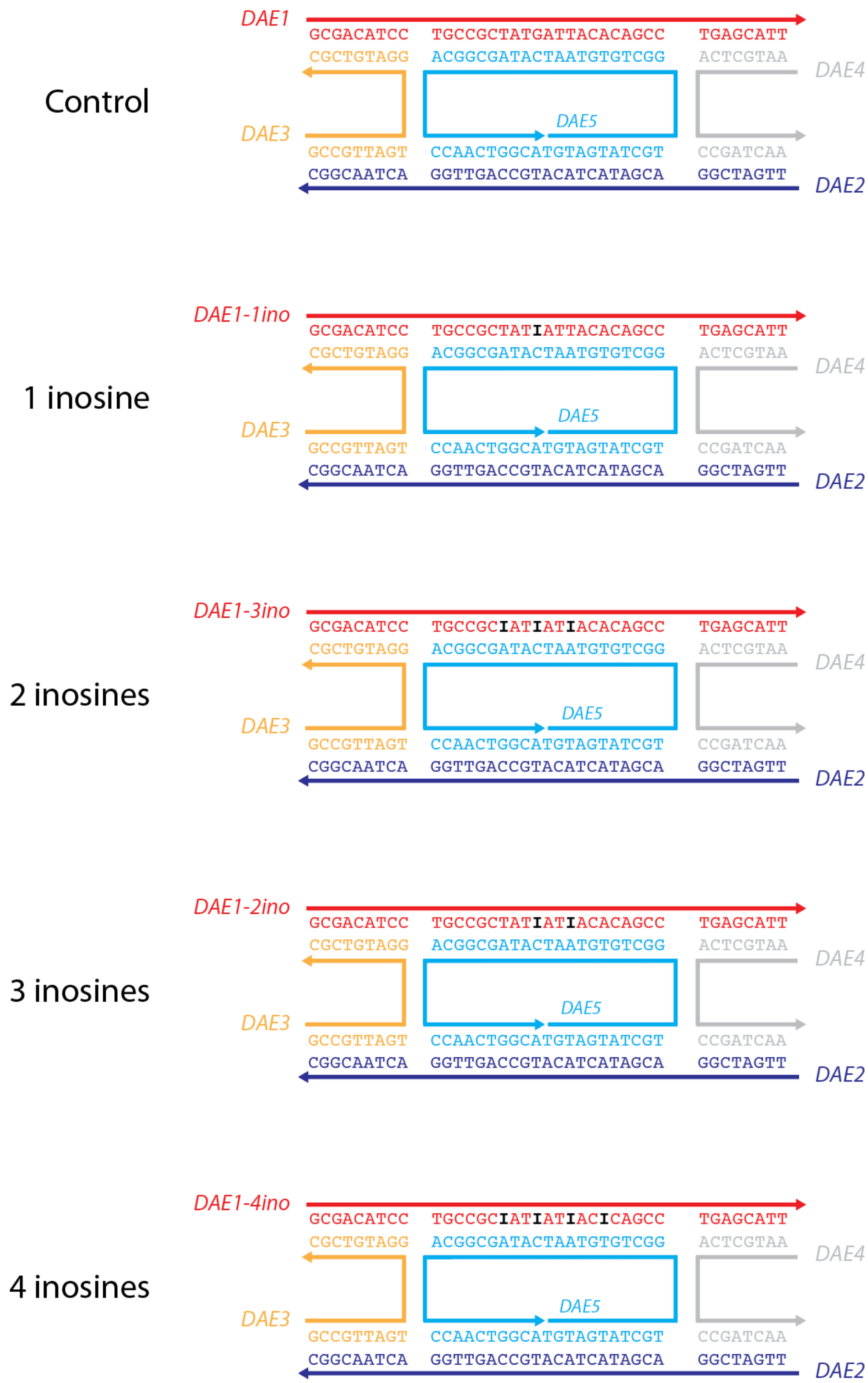

Figure S1. Sequences used in the DX motif. Strand 1 was modified to contain 1-4 inosines.

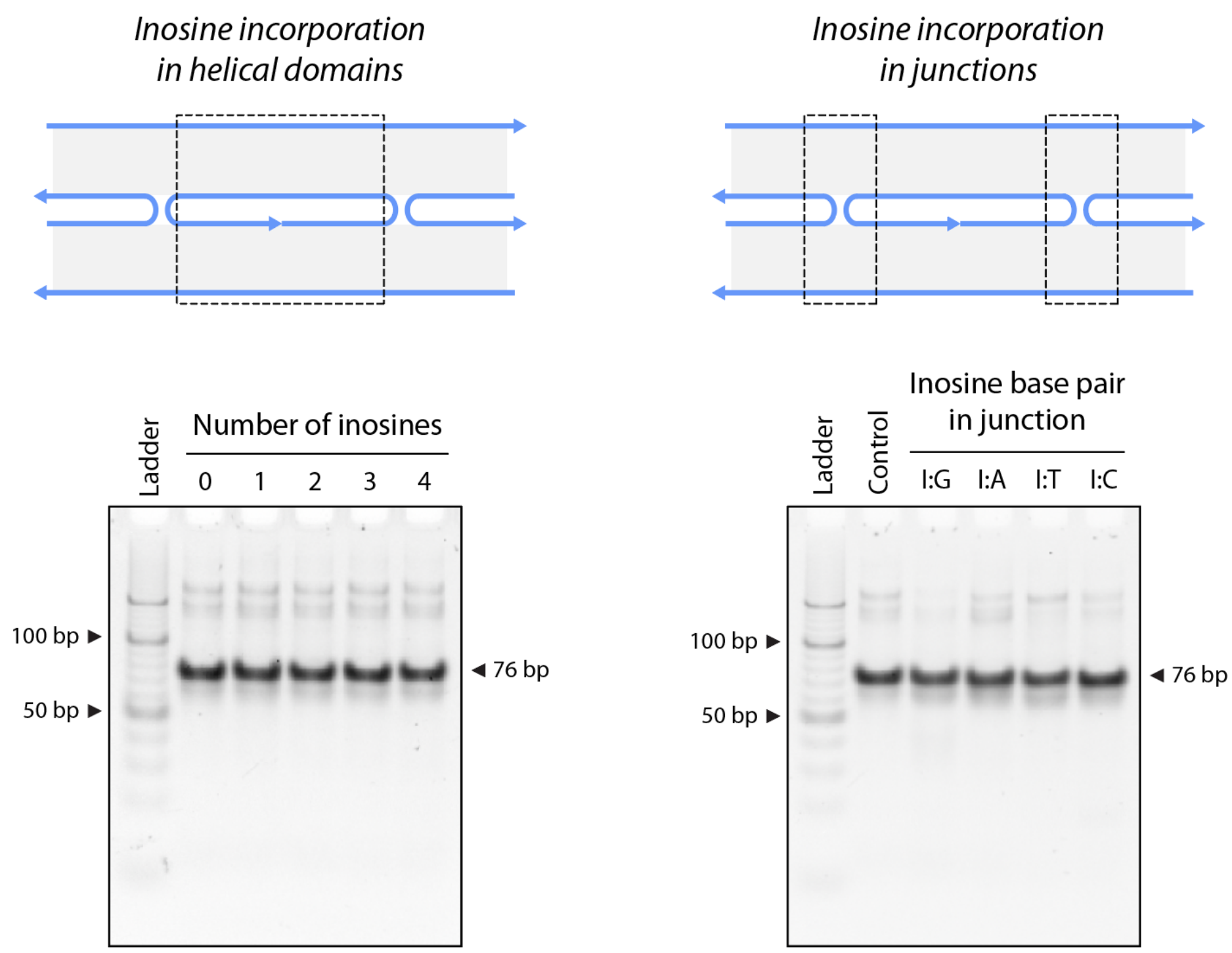

Figure S2. Assembly of DX DNA motif containing inosines. Full images of gels shown in Figure 2. The gels are representative images from replicate experiments.

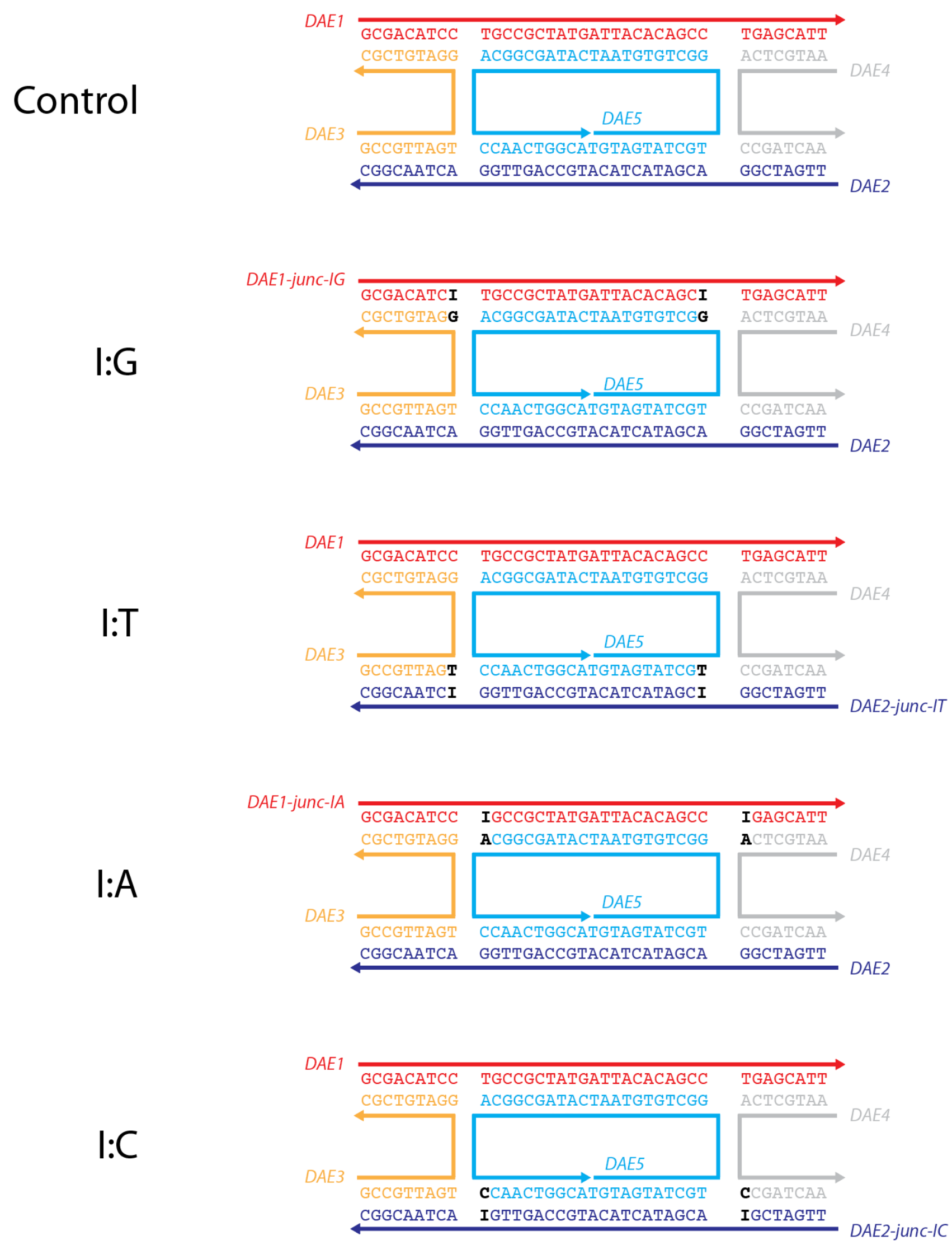

Figure S3. Sequences used in the DX motif, with strands modified to incorporate inosine in the junctions.

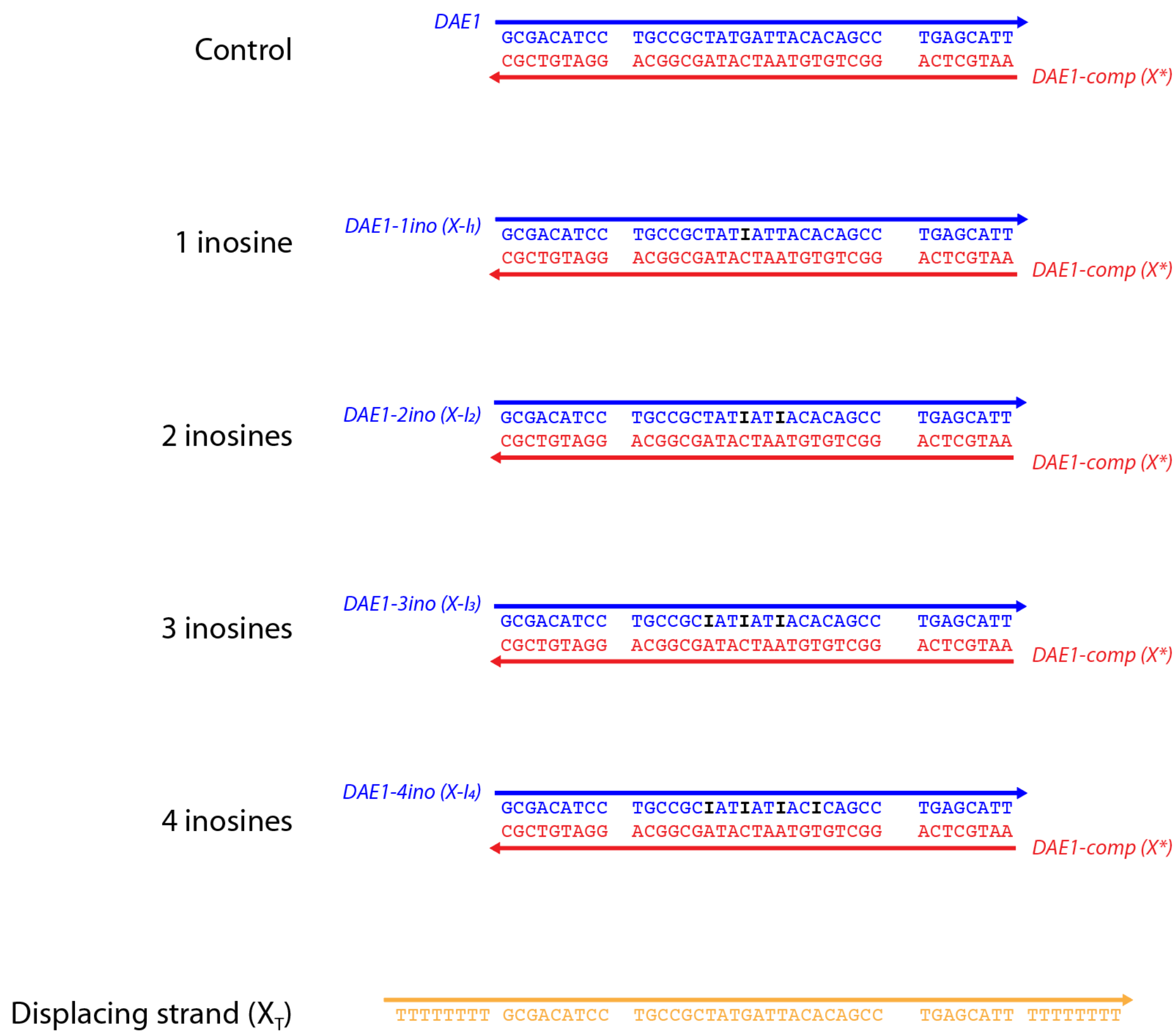

Figure S4. Sequences used in the strand displacement experiments. The displacing strand X_T contained additional Ts at the termini to allow convenient readout by gel electrophoresis.

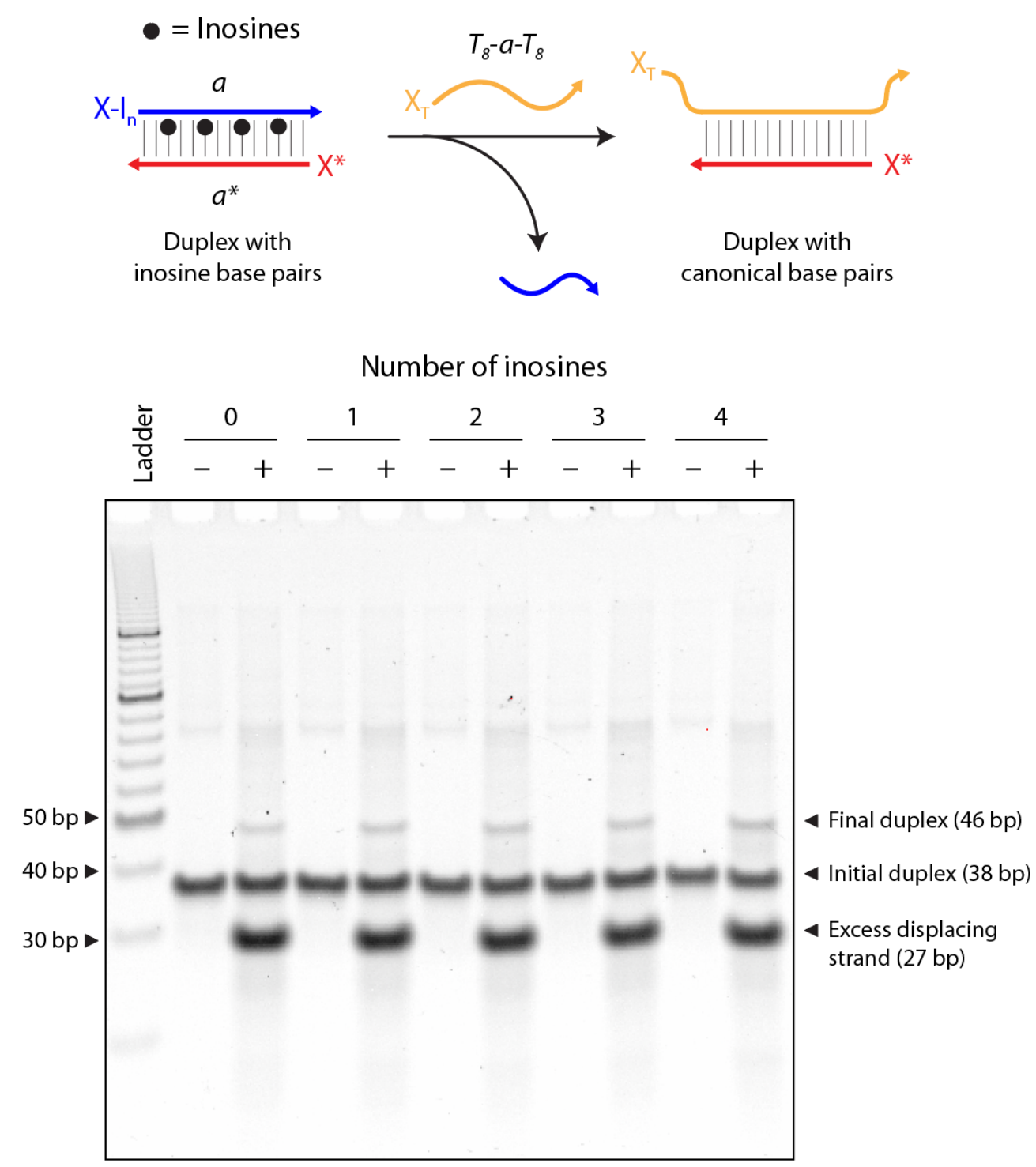

Figure S5. Strand displacement in duplexes containing inosines. Full image of gel shown in Figure 3. The gel is a representative image from replicate experiments.

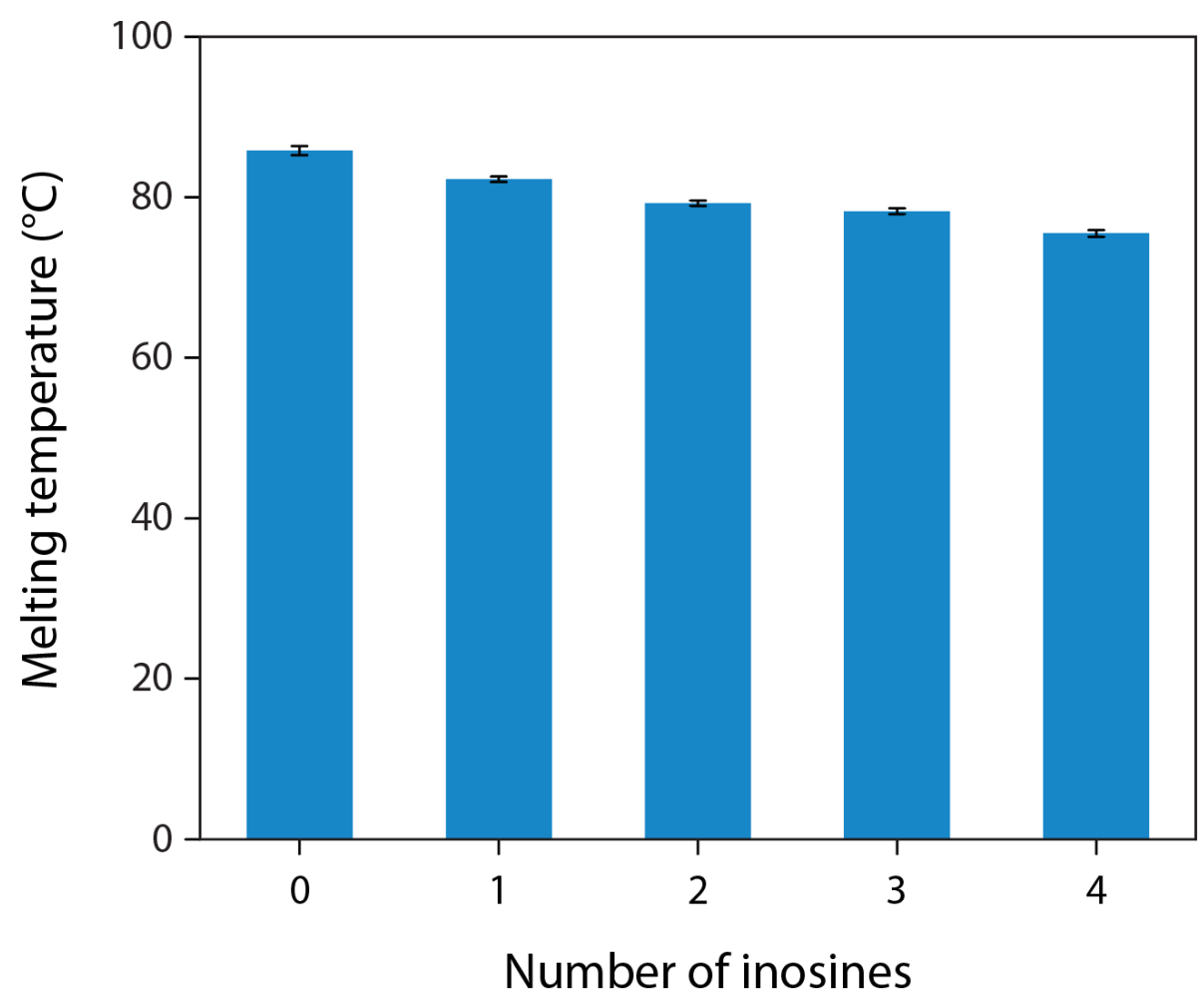

Figure S6. Melting temperatures of duplexes containing inosines.

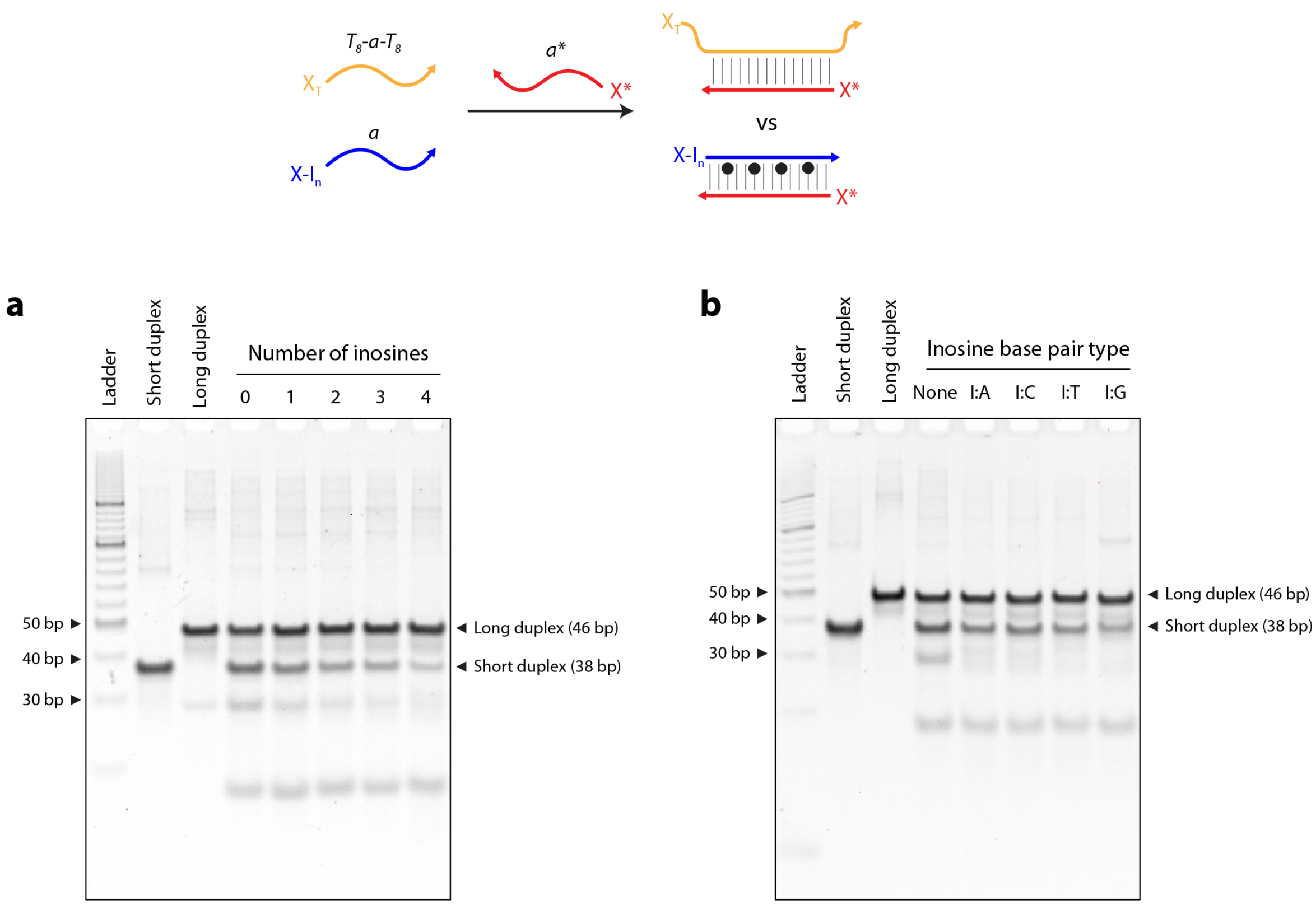

Figure S7. Competition in duplexes containing (a) different number of inosines and (b) different types of inosine base pairs. Full images of gels shown in Figure 3. The gels are representative images from replicate experiments.

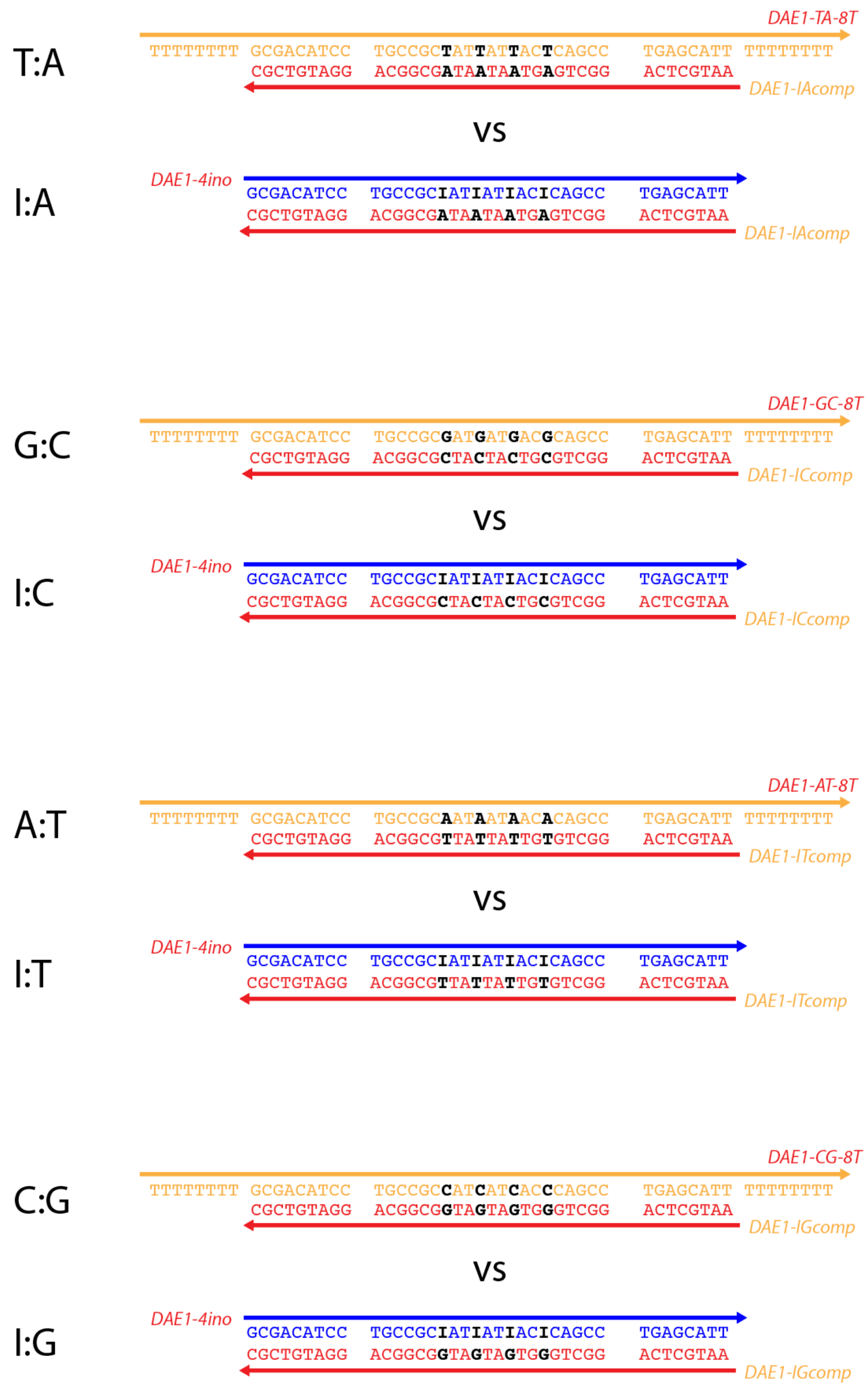

Figure S8. Sequences used in the pre-assembly competition experiments involving different types of inosine base pairs.

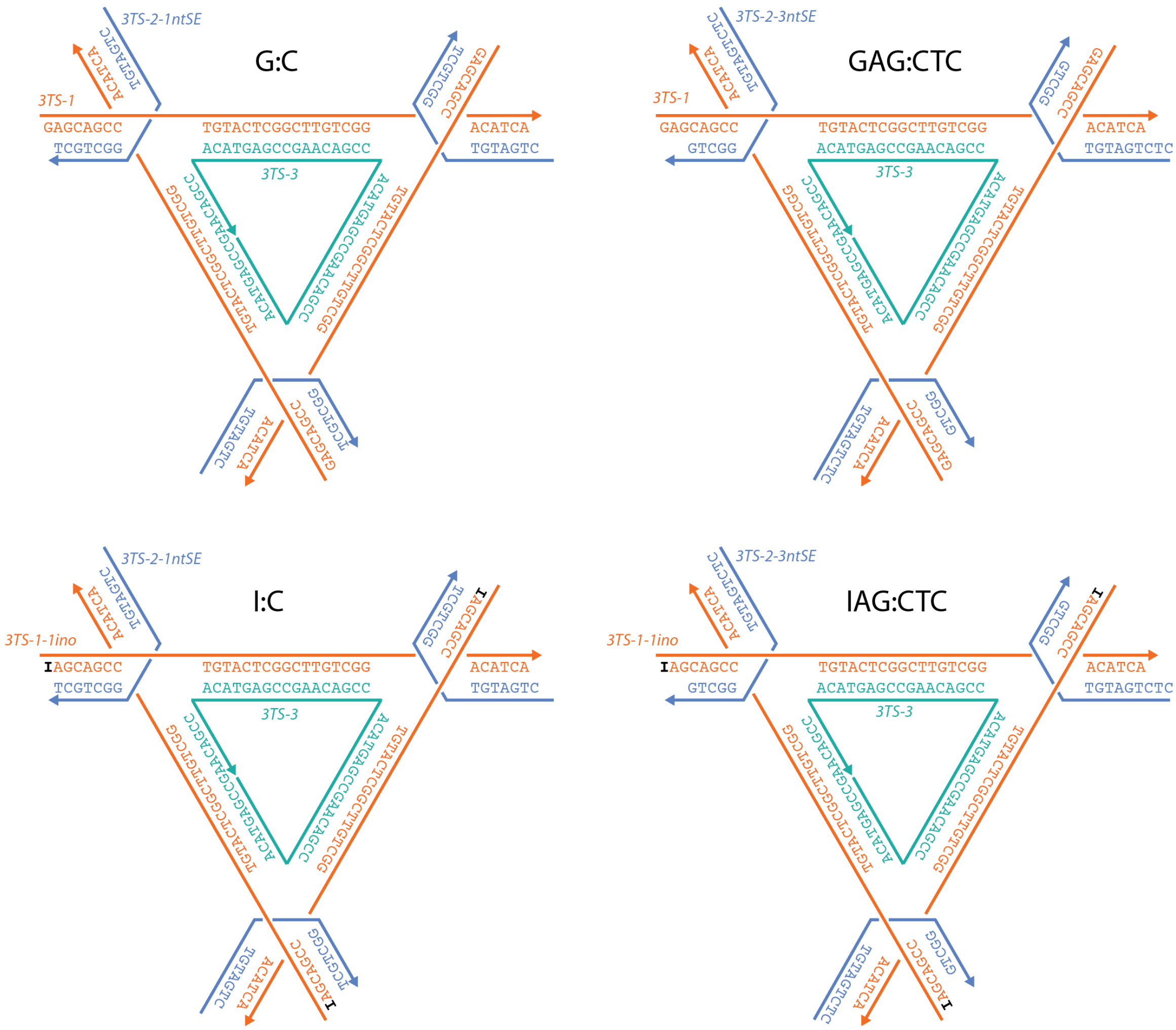

Figure S9. Sequences used in the tensegrity triangle motif. Strand 1 was modified to contain an inosine and strand 2 was modified to obtain different sticky end lengths. The overall length of the duplexes in each edge was constant at 31 bp in all variations.

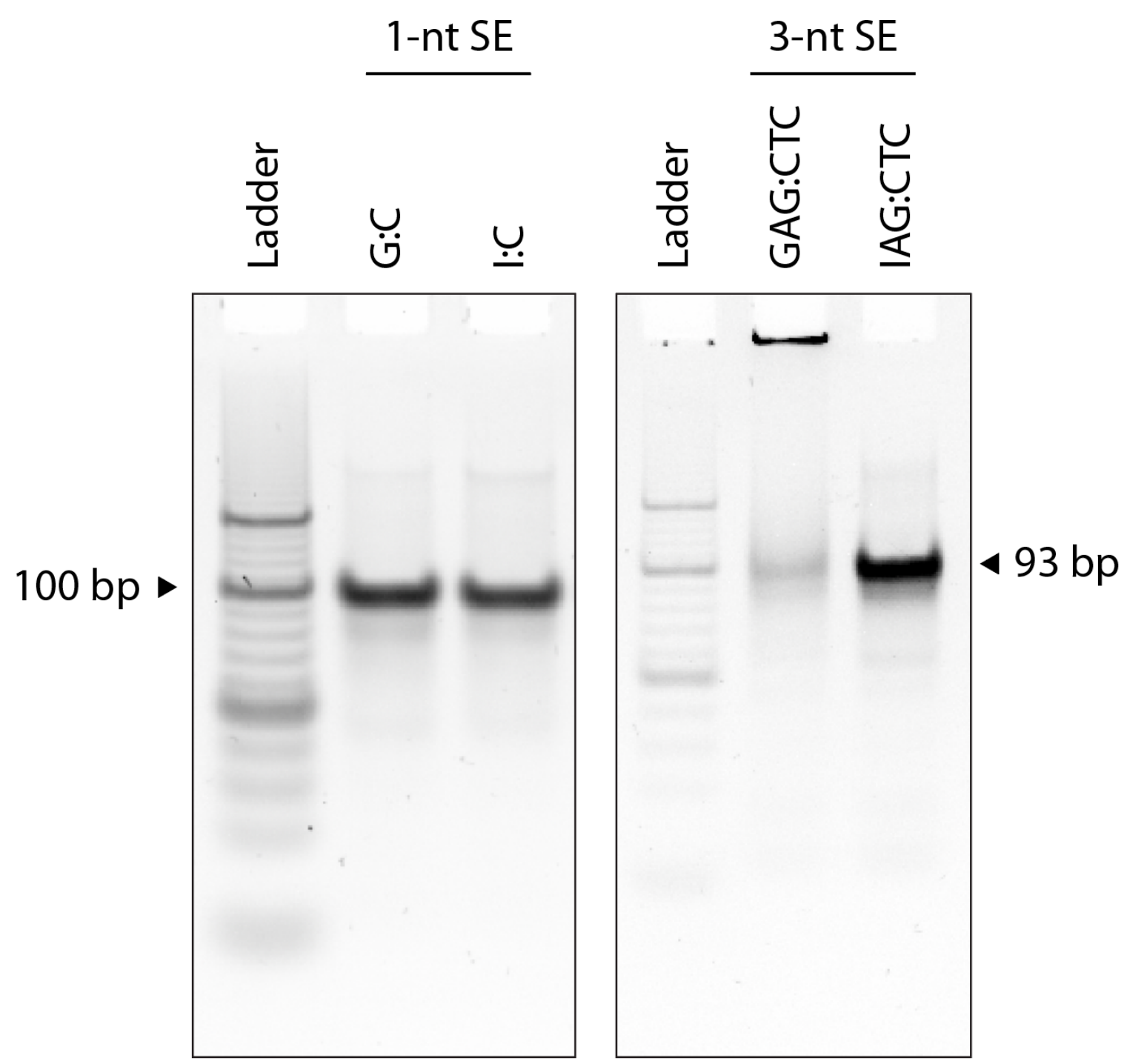

Figure S10. Assembly of the tensegrity triangle DNA motif containing inosines in the sticky ends.

**Double crossover (DX) motif**	
DAE-1	GCGACATCCTGCCGCTATGATTACACAGCCTGAGCATT
DAE-2	TTGATCGGACGATACTACATGCCAGTTGGACTAACGGC
DAE-3	GCCGTTAGTGGATGTCGC
DAE-4	AATGCTCACCGATCAA
DAE-5	TGTAGTATCGTGGCTGTGTAATCATAGCGGCACCAACTGGCA
DAE1-1ino	GCGACATCCTGCCGCTAT**I**ATTACACAGCCTGAGCATT
DAE1-2ino	GCGACATCCTGCCGCTAT**I**AT**I**ACACAGCCTGAGCATT
DAE1-3ino	GCGACATCCTGCCGC**I**AT**I**AT**I**ACACAGCCTGAGCATT
DAE1-4ino	GCGACATCCTGCCGC**I**AT**I**AT**I**AC**I**CAGCCTGAGCATT
DAE1-junc-IG	GCGACATC**I**TGCCGCTATGATTACACAGC**I**TGAGCATT
DAE1-junc-IA	GCGACATCC**I**GCCGCTATGATTACACAGCC**I**GAGCATT
DAE2-junc-IT	TTGATCGG**I**CGATACTACATGCCAGTTGG**I**CTAACGGC
DAE2-junc-IC	TTGATCG**I**ACGATACTACATGCCAGTTG**I**ACTAACGGC
**Duplexes** (used with DAE1 with and without inosines)	
DAE1-comp (X*)	AATGCTCAGGCTGTGTAATCATAGCGGCAGGATGTCGC
DAE1-8T (X_T)	TTTTTTTTGCGACATCCTGCCGCTATGATTACACAGCCTGAGCATTTTTTTTTT
DAE1-IAcomp	AATGCTCAGGCTGAGTAATAATAGCGGCAGGATGTCGC
DAE1-ICcomp	AATGCTCAGGCTGCGTCATCATCGCGGCAGGATGTCGC
DAE1-ITcomp	AATGCTCAGGCTGTGTTATTATTGCGGCAGGATGTCGC
DAE1-IGcomp	AATGCTCAGGCTGGGTGATGATGGCGGCAGGATGTCGC
DAE1-TA-8T	GCGACATCCTGCCGCTATTATTACTCAGCCTGAGCATT
DAE1-GC-8T	GCGACATCCTGCCGCGATGATGACGCAGCCTGAGCATT
DAE1-AT-8T	GCGACATCCTGCCGCAATAATAACACAGCCTGAGCATT
DAE1-CG-8T	GCGACATCCTGCCGCCATCATCACCCAGCCTGAGCATT
**Tensegrity triangle motif**	
3TS1	GAGCAGCCTGTACTCGGCTTGTCGGACATCA
3TS1-1ino	**I**AGCAGCCTGTACTCGGCTTGTCGGACATCA
3TS2-1nt SE	CTGATGTGGCTGCT
3TS2-3nt SE	CTCTGATGTGGCTG
3TS3	CCGAGTACACCGACAAGCCGAGTACACCGACAAGCCGAGTACACCGACAAG

Table S1. Sequences used in this work (written 5′-3′).

DX motif	Melting temperature (°C)
Control	63.5 ± 0.3
1 inosine	63.1 ± 0.1
2 inosines	61.8 ± 0.4
3 inosines	61.7 ± 0.2
4 inosines	60.1 ± 0.6
Junction IG	60.3 ± 0.4
Junction IA	63.3 ± 0.4
Junction IT	61.3 ± 0.4
Junction IC	62.5 ± 0

Table S2. DX motif melting temperatures.

Duplex	Melting temperature (°C)
Control	85.8 ± 0.6
1 inosine	82.3 ± 0.4
2 inosines	79.3 ± 0.4
3 inosines	78.3 ± 0.4
4 inosines	75.5 ± 0.4

Table S3. Duplex melting temperatures.
